# Investigation of Different Types of Biochar on the Thermal Stability and Fire Retardance of Ethylene-Vinyl Acetate Copolymers

**DOI:** 10.3390/polym13081256

**Published:** 2021-04-13

**Authors:** Samuele Matta, Mattia Bartoli, Alberto Frache, Giulio Malucelli

**Affiliations:** 1 Department of Applied Science and Technology and Local INSTM Unit, Politecnico di Torino, Viale Teresa Michel 5, 15121 Alessandria, Italy; samuele.matta@polito.it (S.M.); alberto.frache@polito.it (A.F.); 2 Department of Applied Science and Technology and Local INSTM Unit, Politecnico di Torino, C.so Duca degli Abruzzi 24, 10129 Torino, Italy; mattia.bartoli@polito.it

**Keywords:** EVA, biochar, fire retardance, thermal properties, mechanical behavior

## Abstract

In this work, three biochars, deriving from soft wood, oil seed rape, and rice husk and differing as far as the ash content is considered (2.3, 23.4, and 47.8 wt.%, respectively), were compounded in an ethylene vinyl acetate copolymer (vinyl acetate content: 19 wt.%), using a co-rotating twin-screw extruder; three loadings for each biochar were selected, namely 15, 20, and 40 wt.%. The thermal and mechanical properties were thoroughly investigated, as well as the flame retardance of the resulting compounds. In particular, biochar, irrespective of the type, slowed down the crystallization of the copolymer: this effect increased with increasing the filler loading. Besides, despite a very limited effect in flammability tests, the incorporation of biochar at increasing loadings turned out to enhance the forced-combustion behavior of the compounds, as revealed by the remarkable decrease of peak of heat release rate and of total heat release, notwithstanding a significant increase of the residues at the end of the tests. Finally, increasing the biochar loadings promoted an increase of the stiffness of the resulting compounds, as well as a decrease of their ductility with respect to unfilled ethylene vinyl acetate (EVA), without impacting too much on the overall mechanical behavior of the copolymer. The obtained results seem to indicate that biochar may represent a possible low environmental impact alternative to the already used flame retardants for EVA, providing a good compromise between enhanced fire resistance and acceptable mechanical properties.

## 1. Introduction

Ethylene vinyl acetate (EVA) is a copolymer broadly employed for several applications in different sectors, such as construction, transport, and electrical engineering, as well as biomedical, adhesion, and packaging, among a few to mention [1,2].

Despite its wide uses, EVA is highly inflammable and therefore it has to be protected using specific flame retardant (FR) additives. In this context, so far, several effective flame retardant systems [3,4,5] have been designed and successfully applied to EVA. In particular, over the past 50 years, halogen-based FRs have been considerably employed, providing the copolymer with outstanding flame retardant features. However, the health and environmental hazard derived from the use of halogen-based flame retardants suggested to limit or even stop their utilization both in Europe and the United States [6,7]. In order to overcome this issue, wire and cable industry started to take advantage of the use of inorganic flame retardant fillers for EVA [8,9,10,11].

As an example, El Hage et al. [12] conferred flame retardant feature to EVA by incorporating three new synthesized (namely, aluminum trihydroxyde and two pseudoboehmites) and two commercial hydrated mineral aluminum-based fillers having different aspect ratio (namely, ground milled aluminum trihydroxyde and precipitated boehmite). As assessed by forced combustion tests, pseudoboehmites showed higher flame-retardant features with respect to aluminum trihydroxydes and boehmite, as witnessed by the remarkable lowering of peak of heat release rate values (up to −66% with respect to the unfilled polymer). This finding was ascribed to the high barrier effect exerted by the lamellar-shaped structure of pseudoboehmites.

Lou and co-workers [13] exploited a melt compounding process, incorporating ammonium polyphosphate, aluminum hydroxide, and fluorophlogopite mica into EVA, hence obtaining ceramifiable polymer composites. The obtained results indicated that, among the various EVA composites, those with 1:1 ratio of ammonium polyphosphate:aluminum hydroxide exhibited the best flame retardance (LOI value achieved 29.7%; peak of heat release rate and total heat release decreased by 81 and 51%, respectively) and the highest char residues.

Moradkhani et al. [14] prepared EVA-based composites containing exfoliated graphite nanoplatelets grafted by ammonium polyphosphate. The composites containing 30 wt.% of modified nanoplatelets revealed a significant decrease of peak of heat release rate (−63%) and total heat release (−32%), owing to the formation of a compact and cohesive char layer. Besides, these composites limited the decrease of ductility as a consequence of the incorporation of the filler.

Very recently, Xu et al. [15] exploited the combination of aluminum hydroxide and melamine cyanurate for conferring flame retardant properties to EVA. In particular, the composite containing 100 phr of EVA, 60 phr of aluminum hydroxide and 40 phr of melamine cyanurate exhibited the highest limiting oxygen index (LOI) value (27.5%) and V-0 rating in vertical flammability tests.

In the last ten years, the seeking for flame retardant systems showing acceptable effectiveness and low environmental impact, and matching the up-to-date circular economy concept has strongly motivated the scientific community toward the design and development of new products never utilized for flame retardant purposes: in particular, different systems derived from bio-sources, and even wastes or scraps from the agro-food industry have been identified and successfully applied to different polymeric matrices and fabrics [16,17,18]. In this context, very recently, biochar (BC), a solid product derived from the thermo-chemical conversion of biomasses in an oxygen-limited environment, is gaining great interest as a cheap carbon source; in particular, so far, it has been employed in the field of environmental management [19], agriculture [20], and in materials science [21], because of its extremely stable honeycomb-like carbonaceous structure. This latter also suggests a possible use of biochar for designing flame retardant systems suitable, alone or in combination with the standard flame retardant additives, for improving the fire resistance of different polymer systems upon application of an irradiative heat flux or exposure to a flame. As there are only a few scientific papers dealing with this interesting potential use [22,23,24,25], the present work aims at further unveiling its flame retardant potential in an EVA copolymer. To this aim, in this work we selected three different types of biochar, obtained from different bioproducts (namely soft wood, oil seed rape, and rice husk) and exhibiting an increased ash content (2.3, 23.4, and 47.8 wt.%, respectively). These fillers were incorporated at different loadings, ranging from 15 to 40 wt.% in an ethylene vinyl acetate copolymer (vinyl acetate content: 19 wt.%), using a co-rotating twin-screw extruder. The resulting compounds were thoroughly characterized as far as their thermal, mechanical and flame retardant properties are considered.

## 2. Materials and Methods

### 2.1. Materials

EVA (melt flow rate of 12 g/10 min at 190 °C/2.16 kg, density of 0.942 g/cm^3^ and vinyl acetate content of 19 wt.%) was kindly supplied by Versalis SPA under commercial name Greenflex MQ40, a grade for injection molding and compounding.

The selected biochars were purchased from UK Biochar Research Centre and were produced by pyrolytic treatment of mixed softwoods, oil seed rape, and rice husk, using a pilot-scale rotary kiln unit [26], setting the highest treatment temperature to 550 °C. Each resulting material was further annealed at 1000 °C by using a vertical furnace and a quartz reactor, at a heating rate of 50 °C/min, and kept at the final temperature (1000 °C) for 30 min in argon atmosphere.

Afterwards, the annealed biochars were further pulverized by grinding with a mechanical blender (Savatec BB90E, Turin, Italy) for 10 min and ball-milled for 10 min. Their ash content was evaluated by incineration at 550 °C for 6 h, using a static furnace. In Table 1, the labels of the samples with a brief recap on the production conditions are reported.

### 2.2. Preparation of the Compounds

**Figure 1:**
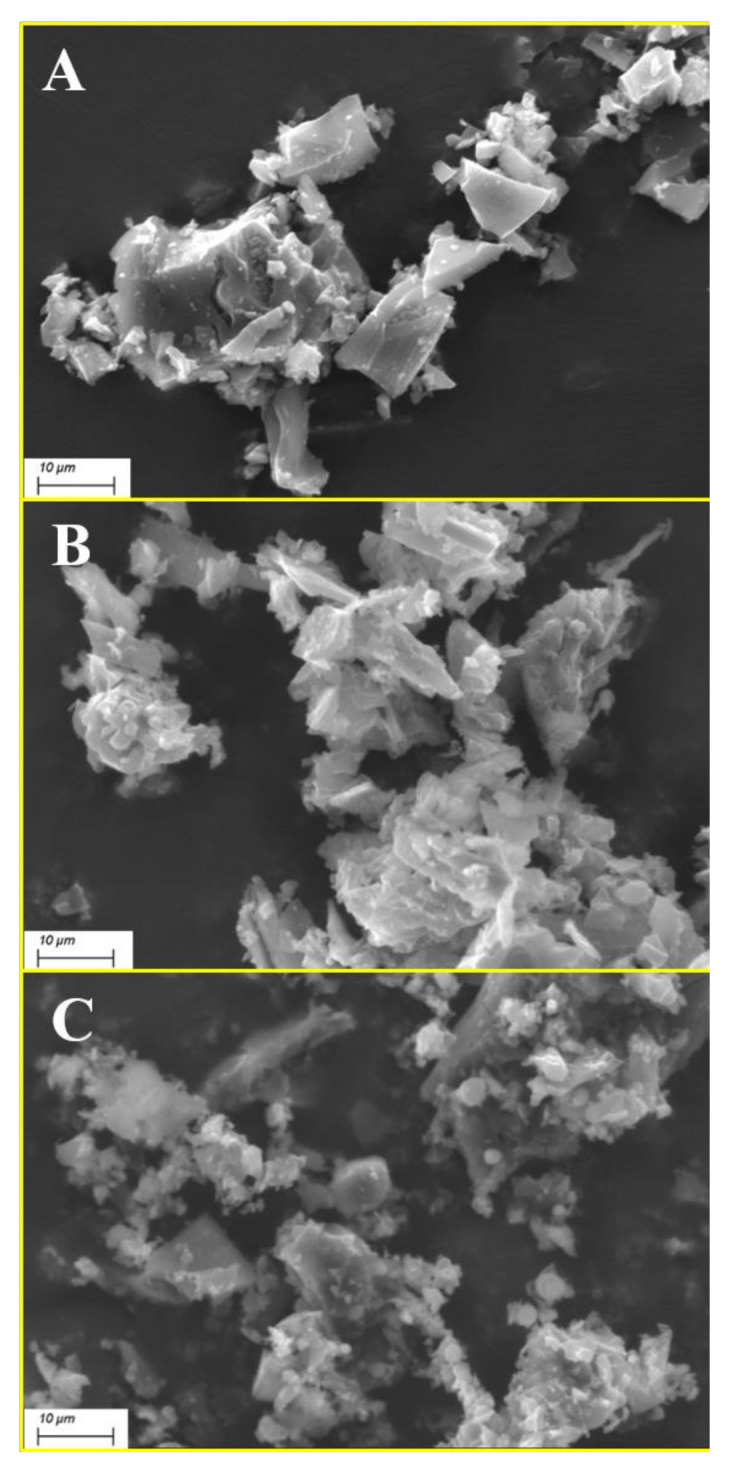
SEM images at 3000× magnification of biochar (BC) low (**A**), BC medium (**B**), and BC high (**C**).

The compounds were processed in a co-rotating twin-screw extruder *Process 11* from ThermoFisher Scientific (Waltham, MA, USA) with a screw diameter of 11 mm and L/D ratio of 40. The extruder was divided into 8 blocks and a die with a single hole heated with the temperature profile reported in Figure S1. Two volumetric feeders dosed the EVA pellets on the first block of the extruder and BC powders on the third block. The block number seven had a window for degassing and the die was equipped with a pressure sensor and a thermocouple for recording the temperature of the melt (~145 °C and 25–30 bar, respectively). The screw profile is shown in Figure S1 and was chosen for maximizing the transfer of mechanical energy in order to optimize the dispersion of BC. A typical melting zone composed by transport elements was used for melting the polymer. To provide proper mixing of BC and molten polymer, as well as to reduce the size of agglomerates or large particles, three kneading blocks were inserted. Every single element of the kneading blocks had a chosen orientation with respect to the next one of 30°, 60°, and 90°, as shown in Figure 1. The speed of the extruder was 500 rpm and the mass flow of the EVA feeder was fixed at 350 g/h, while the powders feeder was set to obtain the required filler loading. The processed material flowed out from the extruder inside a water batch for a quick cooling and then it was pelletized. Pellets obtained by extrusion were then processed, obtaining specimens suitable for the characterization tests.

In particular, cone calorimetry samples were prepared using a Collin P200T press (Maitenbeth, Germany) with hot plates for compression molding. Square samples (100 × 100 × 3 mm^3^) were obtained at 150 °C and 100 bar. The same method was exploited for molding specimens (125 × 16 × 3 mm^3^) for flammability tests.

Specimens with “dog bone” shape for mechanical tests (75 × 4 × 2 mm^3^) were obtained using a Babyplast 610P Standard injection molding machine, from CRONOPLAST SL (Abrera, Spain), operating at 150 °C, first injection time of 8 s at 85 bar for filling the mold and second injection time of 25 s at 65 bar of maintenance, with a total time cycle of 45 s.

### 2.3. Characterization Techniques

BC samples were analyzed through Raman spectroscopy by using a Renishaw inVia apparatus (H43662 model, Gloucestershire, UK) equipped with a green laser line (514 nm) with a 50× objective. Raman spectra were recorded in the range between 250 cm^−1^ and 3500 cm^−1^.

The morphologies of biochar powders and EVA/BC composites were investigated using an EVO 15 scanning electron microscope (SEM) from Zeiss (Oberkochen, Germany), coupled to Ultim Max 40 energy dispersive X-ray (EDX) micro-analyzer by Oxford Instruments (High Wycombe, UK), with AZtecLive integrated software. Fragments of the compounds obtained by a brittle fracture in liquid nitrogen were fixed to conductive adhesive tapes and gold-metallized.

Differential scanning calorimetry (DSC) analyses were carried out using a Q20 TA Instrument apparatus (New Castle, DE, USA), on samples of about 8 mg, placed in sealed aluminum pans. All the materials were first heated up under dry nitrogen from 30 to 150 °C at 10 °C/min, in order to erase the previous thermal history. Then, samples were cooled from 150 °C to 30 °C at 10 °C/min and a second heating was applied up to 150 °C at 10 °C/min. The crystallization temperature (T_c_), crystallization enthalpy (ΔH_c_), melting temperature (T_m_), and melting enthalpy (ΔH_m_) were identified, using the 2nd heating up trace. The crystallinity degree (Χ_c_) of the unfilled polymer and of the compounds was calculated using the following equation (Equation (1)):

(1) $$X_{c}=\frac{{\Delta H}_{m}}{{\Delta H}_{100}\;\left(1-x\right)\;}*100$$

where x is the filler weight percentage and ΔH_100_ represents the melting enthalpy of the 100% crystalline polymer matrix, equal to 290 J/g [27] (since it is assumed that crystallization occurs in polyethylene segments only).

The thermal and thermo-oxidative behavior of BC powders and EVA/BC composites was investigated by thermogravimetric analysis (TGA) using a Q500 system from TA Instrument (New Castle, DE, USA); the samples were heated from 30 to 700 °C at 10 °C/min, in nitrogen or air (gas flow: 60 mL/min). The tests were performed placing about 10 mg of sample in open alumina pans. T_onset_ (temperature of starting degradation), T_max_ (temperature, at which maximum weight loss rate is observed in dTG - derivative - curves) and residue at 700 °C were measured.

Flammability tests were performed in vertical configuration on EVA and EVA/BC compounds according to the ASTM D3801 standard. The specimen was held in vertical position by a metallic clamp and was ignited at the bottom using a 20 mm methane flame, tilted at 45°. The flame was applied twice for 10 s and the after flame times were recorded. A cotton wad was positioned 30 cm under the bottom down of specimen, for evaluating the incandescent dripping. Times of after-flame burning (t_1_ and t_2_), the evolution of fire and melt dripping were registered.

The combustion behavior was investigated through cone calorimetry tests according to the ISO 5660 standard, using a Fire Testing Technology (West Sussex, UK) instrument. Square samples (50 × 50 × 3 mm^3^) were exposed to a 35 kW m^−2^ heat flux in horizontal configuration. The specimens were wrapped in aluminum foil except the upper face and placed on a load cell, maintaining 25 mm between surface and cone heater. For each formulation, the test was repeated three times and the results were averaged. Time to ignition (TTI, s), peak of heat release rate (pHRR, kW m^−2^), time to peak (s), total heat release (THR, kW m^−2^), total smoke release (TSR, m^2^/m^2^), fire performance index (FPI, (kW/m^2^)/s), fire growth rate index (FIGRA, (kW/m^2^)/s), and final residue were evaluated.

Tensile tests were performed using a Instron 5966 dynamometer (Norwood, MA, USA), following the ASTM D638-03 standard. The tests were carried out using a 5kN load cell, at 1 mm/min rate until 0.2% deformation was reached; then, the rate was increased up to 50 mm/min until the specimen broke. Five specimens were tested for each formulation and the average values of the tensile modulus (E), the elongation at break (ε), the tensile strength (σ_y_) were calculated.

## 3. Results

### 3.1. Subsection

SEM observations were performed both on biochar powders and on EVA/BC compounds in order to assess the morphology of different types of fillers as well as their distribution within EVA. Furthermore, EDX micro-analyses were performed on BC powders in order to evaluate the chemical composition of the fillers.

Figure 1 shows some typical images of BC fillers: their morphology is in the micro scale, specifically between few micrometers up to 30–40 µm, with irregular shapes. The elemental analysis carried out by energy dispersive X-ray spectroscopy (Figure S2) confirmed the presence of C and O as main elements for all the investigated biochar types as expected. Other relevant elements identified are listed as follows:

1. Ca (0.5 wt.%) and K (0.5 wt.%) for BC low;
2. K (4.4 wt.%), Si (2.5 wt.%) and Ca (1.3 wt.%) for BC medium;
3. Si (4.2 wt.%), K (3.9 wt.%), P (3.1 wt.%) and Mg (1.1 wt.%) for BC high.

On the basis of the carbon percentage detected by EDX analysis, it is reasonable to assume that the inorganic content was accumulated into the core of the particles, also considering a depth resolution for this technique around 1 μm [28].

Nonetheless, it was quite remarkable that the carbon content (wt.%) of BC high is close to 70% even after the treatment at 1000 °C, while it approaches 90% in the case of BC low. All the three types of biochar underwent a thermal annealing to remove as much as possible the surface residual groups for a better understanding of the ash role on the properties of the resulting composites [29].

**Figure 2:**
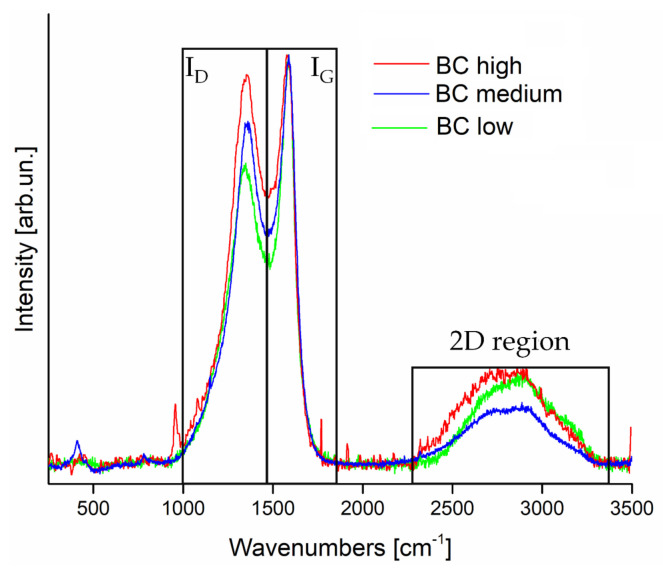
Raman spectra of BC low (green), BC medium (blue), and BC high (red) in the range from 250 cm^−1^ to 3500 cm^−1^. Spectra are normalized to I_G_ peak.

The Raman spectra of three biochars (Figure 2) showed materials that are laying on the transition to graphitic carbon but still highly disordered [30], as already reported in the scientific literature [31]. In particular, BC low, BC medium, and BC high displayed I_D_/I_G_ values of 1.3, 1.5, and 1.7 respectively. These values are in good agreement with those previously reported for biochar produced at 1000 °C [32]; besides, they show that the low inorganic content of BC low allowed the formation of more ordered carbon structures.

2D region was poorly resolved showing materials far from a complete reorganization in graphitic like structures. As reported by several studies [31,33], a temperature higher than 1300 °C is required to display a narrow bands in the region from 2500 cm^−1^ up to 3500 cm^−1^.

**Figure 3:**
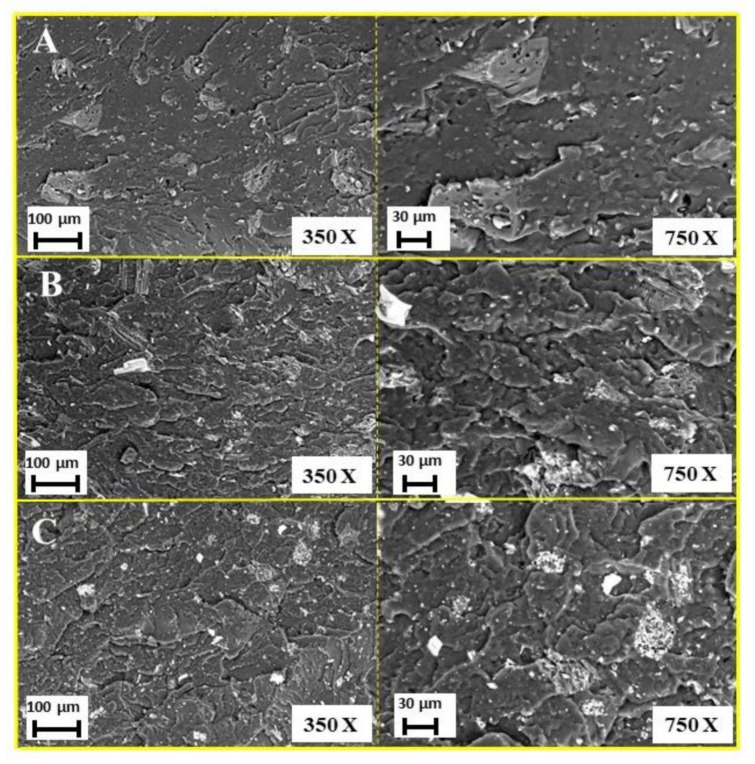
SEM images at different magnification (350× and 750×) of EVA + 20 wt% of: BC low (**A**); BC medium (**B**); and BC high (**C**).

As an example, the typical SEM images of the compounds containing 20 wt.% of the three biochars are presented in Figure 3. Particles are well distributed within the polymer matrix and their size is substantially micrometric, though some bigger aggregates (not exceeding 100 microns) are clearly visible. These findings indicate that the experimental conditions adopted for compounding were suitable for obtaining a homogeneous dispersion of the fillers, regardless of their type and loadings.

### 3.2. DSC Analyses

Table 2 collects the main DSC data recorded during the selected thermal cycles for EVA and its compounds. First of all, it is worthy to note that the presence of BC, irrespective of its type and loading, does not affect the melting temperature (T_m_) of the composites, which is almost unchanged with respect to unfilled EVA (about 85 °C). Besides, T_c_ is practically unaffected by the presence of the filler, as it slightly increases by about 3 °C, regardless of the biochar type and loading. Conversely, the degree of crystallinity (X_c_) for all the compounds decreases in the presence of BC, which seems to slow down the crystallization process: this effect is further enhanced by increasing the filler loading.

### 3.3. Thermogravimetric Analyses

**Figure 4:**
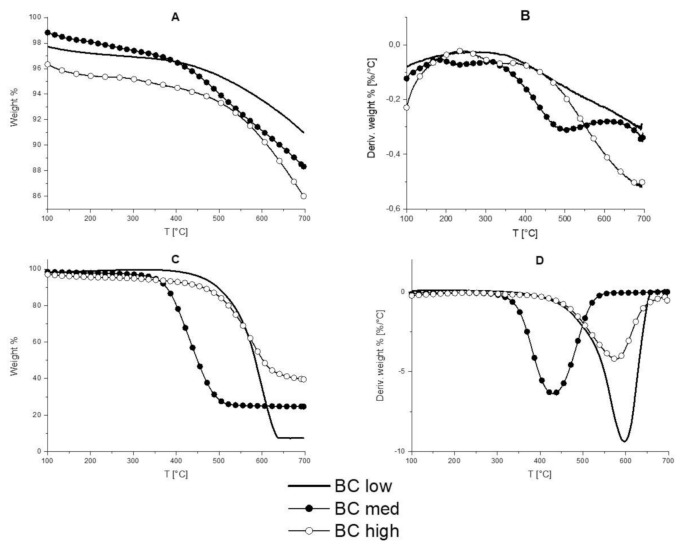
Thermogravimetric and dTG curves in nitrogen (**A**,**B**) and air (**C**,**D**) of biochar low, medium and high.

Thermogravimetric analyses were performed in order to evaluate the thermal and thermo-oxidative stability of both BC powders and their composites. Figure 4 shows the thermograms for BC powders in nitrogen and air; the obtained data are summarized in Table S1.

**Figure 5:**
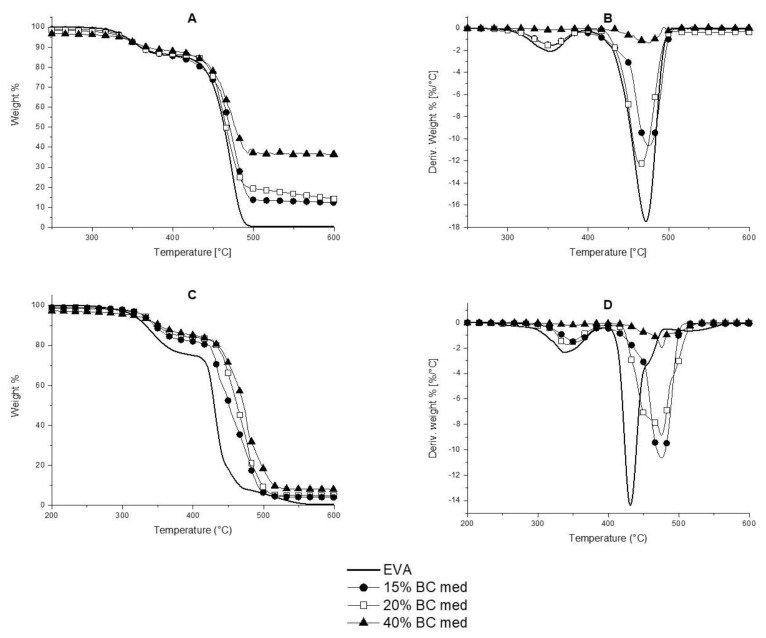
TG and dTG curves of EVA and its compounds containing BC medium in nitrogen (**A**,**B**) and in air (**C**,**D**).

Unlike in nitrogen, where the differences among the different types of BC are almost negligible, in air atmosphere (Figure 5C), the different BC powders show a single step degradation, with significant differences of T_onset_ values. More specifically, BC low is the filler that starts degrading at the highest temperature (T_onset_ is about 513 °C), while BC medium and BC high start degrading at 365 and 487 °C, respectively (Table S1). Besides, different residues at 700 °C are obtained for BC low, medium, and high, namely 7.4, 24.6, and 39.6%, respectively: these data are in agreement with the ash content values of the three carbon products.

Figure 5 shows the typical TG thermograms for EVA and for the BC medium compounds; Table S2 collects the obtained data. In nitrogen, EVA shows a two-steps degradation. The first step, occurring between 300 °C and 390 °C, is attributed to the acetic acid evolution, i.e., the mass loss is proportional to the quantity of acetate groups initially present in the copolymer. The second decomposition stage (occurring between 405 °C and 500 °C) is attributable to the chain scission phenomena [34]. When the different types of BC are incorporated into EVA, apart from a slight increase of T_onset_ values, which may be ascribed to a weak protection effect exerted by the fillers, it is worthy to note that the latter contribute to the increase of the residue at 700 °C with respect to the unfilled copolymer.

In air, EVA decomposes according to three consecutive steps. The third decomposition can be ascribed to the oxidation of the products formed during the previous steps. The incorporation of the different BC powders increases the T_onset_, T_max1_, and T_max2_ values, hence indicating a protection effect exerted by the fillers: the highest increase of the aforementioned temperatures is observed for BC medium-containing composites. Unlike oil seed rape (BC medium), rice husk (BC high) accumulates more silicon in its tissues [35], in form of silica structures known as phytoliths [36]. These structures display a great amount of Si-OH sites, suitable for interacting with both polysaccharides and glycoproteins [37], which could be preserved after the pyrolysis processes. These silica acidic structures may accelerate the thermo-oxidative degradation of EVA-BC high compounds [38], hence determining a lower stability. 

In addition, the presence of the BC powders allows increasing the residues at the end of the tests. Therefore, both the type of inorganic components and their amount in the selected biochars seem to play a relevant role in the thermal and thermo-oxidative stability of the prepared compounds.

### 3.4. Flammability Tests

Vertical flame spread tests were performed on EVA and on its compounds containing the different biochars; the results are listed in Table S3. The tests proved the V-2 classification of the specimens with 15 and 20 wt.% BC, without showing any improvement provided by the incorporation of the fillers with respect to the unfilled EVA. On the contrary, the compounds with 40 wt.% of BC were not classified (NC) for this type of test. Indeed, the specimens were not able to extinguish the flame after the second flame application, and the flame reached the metallic clamps failing the test. This behavior could be explained by the high thermal conductivity given to the samples by the presence of high quantity of BC in 40 wt.% BC compounds.

### 3.5. Forced-Combustion Behavior

**Figure 6:**
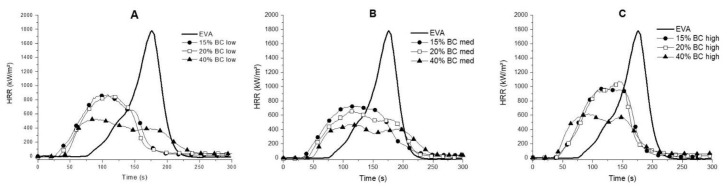
HRR vs. time cone calorimetry curves of EVA and its compounds: EVA BC low (**A**); EVA BC med (**B**); and EVA BC high (**C**).

Cone calorimetry tests were performed on EVA and its compounds using a standard irradiative heat flux of 35 kW m^−2^. The thermal and smoke parameters recorded during the tests are listed, respectively, in Table 3 and Table 4; Figure 6 shows the HRR vs. time curves and Figure S3 displays the residues at the end of the tests.

From an overall point of view, it is worthy to note that the incorporation of the different BC fillers anticipates the ignition of the samples (i.e., decreases TTI values, which, in turn, increase with increasing the filler loading) and lowers both the thermal and smoke parameters. In particular, pkHRR values of the composites are significantly lowered in the presence of the different BC powders; the highest decrease is observed when BC medium is incorporated into EVA. Indeed, for samples loaded with 40 wt.% of BC medium, the pkHRR values decrease by 74% as compared to the unfilled EVA. As a consequence of pkHRR decrease, also FPI (pkHRR/TTI ratio) and FIGRA (pkHRR/time to peak) show a remarkable lowering for the samples containing the BC medium. THR values significantly decrease for the compounds containing BC low and medium and the residues after tests are remarkably increased: all these findings are clear evidences of the formation of a stable char, which protects the polymer matrix from the exposure to the irradiative heat flux. Finally, from an overall point of view, the incorporation of BC promotes the decrease of TSR and SEA values, as shown in Table 4.

### 3.6. Mechanical Behavior of EVA and Its Compounds

**Figure 7:**
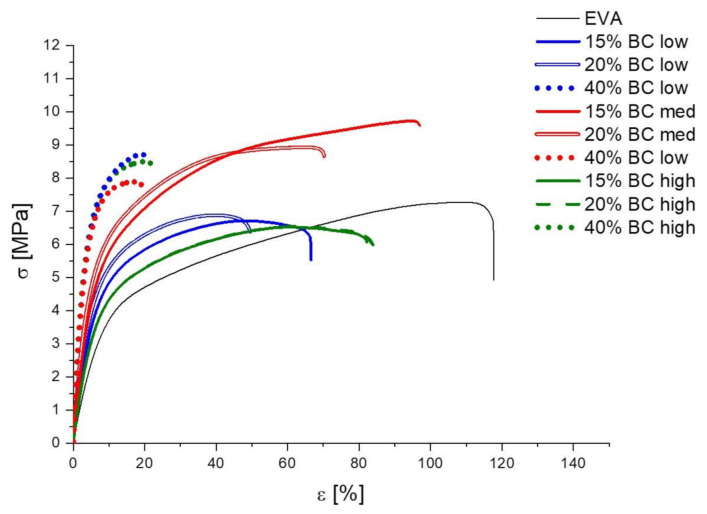
Stress–strain curves for EVA and EVA/BC compounds.

In order to assess the effect of the presence of the biochars on the mechanical behavior of EVA, tensile tests were carried out on EVA and its composites. Figure 7 shows the typical stress–strain curves obtained; Table 5 collects the results in terms of tensile modulus, elongation at break, and tensile strength. As expected, the incorporation of BC, irrespective of the type and content, increases the stiffness and lowers the ductility of EVA copolymer, though ductility is significantly decreased only at the highest filler loading (i.e., 40 wt.%). Besides, the reinforcing effect exerted by the carbonaceous filler is well documented by the tensile modulus increase: in particular, this reaches the values of 221.1 MPa for compound containing 40 wt.% of BC high, i.e., more than four times higher than that of unfilled EVA (E = 50 MPa).

## 4. Conclusions

In this work, the thermal, mechanical, and fire behavior of an EVA copolymer compounded with increasing loadings of three different selected biochars was thoroughly investigated. First of all, regardless of the type and ash content, the incorporation of the bio-sourced fillers turned out to decrease the crystallization ability of the copolymer (by about 47% for the highest filler loading of BC high). The high thermal conductivity of the carbon-based fillers was detrimental as far as the resistance to the propagation of the flame was considered: in fact, all the tested samples were V-2 rated or, at the highest BC loadings, not classifiable. Conversely, the incorporation of the biochars into EVA showed some enhancements in forced-combustion tests, which revealed a remarkable decrease of both thermal and smoke parameters, demonstrating the thermal shielding effect provided by the selected fillers and their behavior as effective smoke suppressants. In particular, pkHRR values were lowered by about 65, 74, and 70% when BC low, BC medium, and BC high were incorporated into the copolymer at the highest loading, respectively. Besides, 40 wt.% of BC medium allowed decreasing the THR by about 27%. Finally, as expected, the biochars were responsible for the increase of the stiffness (by more than four times as compared with the unfilled EVA) and the decrease of ductility (up to about −80% when 40 wt.% of any biochar was incorporated into the copolymer) of the compounds, highlighting the reinforcing effect exerted by the selected fillers.

## Figures and Tables

**Table 1:** List of codes used to identify biochar samples.

Code	Source	Treatment Temperature	Ash Content (%)
BC low	Soft wood	1000 °C	2.3 ± 0.8
BC medium	Oil seed rape	1000 °C	23.4 ± 1.1
BC high	Rice husk	1000 °C	47.8 ± 1.3

**Table 2:** Results from DSC analysis for ethylene vinyl acetate (EVA) and its compounds.

Sample	T_c_ (°C)	Xc (%)	ΔH_c_ (J/g)	T_m_ (°C)	ΔH_m_ (°C)
EVA	62.7	4.5	27.6	85.3	13.2
15% BC low	65.3	3.7	19.6	85.5	9.0
20% BC low	65.0	3.6	17.1	85.3	8.4
40% BC low	66.1	3.2	12.9	84.4	5.6
15% BC med	65.4	3.6	20.5	85.0	8.8
20% BC med	65.1	3.5	19.1	85.0	8.2
40% BC med	65.9	3.0	13.9	84.9	5.2
15% BC high	65.5	3.2	17.1	85.6	7.9
20% BC high	65.5	3.1	15.8	85.2	7.2
40% BC high	65.5	2.4	14.6	85.1	4.2

**Table 3:** Main thermal parameters by cone calorimetry tests.

Specimen	TTI [s]	pkHRR [kW m^−^^2^]	pkHRR Reduction [%]	Time to Peak [s]	THR [MJ m^−^^2^]	Residue Mass [%]	FPI $\left[\frac{\left(kW/m^{2}\right)}{s}\right]$	FIGRA $\left[\frac{\left(kW/m^{2}\right)}{s}\right]$
EVA	74	1803.0	-	178	96.6	0	24.8	10.1
15% BC low	24	883.9	51	104	85.7	7	37.6	8.5
20% BC low	27	862.5	52	112	80.3	8	32.5	7.7
40% BC low	35	623.9	65	111	76.8	27	17.9	5.9
15% BC med	34	742.3	59	131	90.8	8	22.2	5.8
20% BC med	36	660.9	63	117	83.8	10	18.7	5.6
40% BC med	45	478.1	74	113	70.4	18	10.7	4.2
15% BC high	37	1008.2	44	114	95.8	10	27.3	8.9
20% BC high	39	1030.2	43	139	90.5	8	26.8	7.4
40% BC high	39	534.2	70	87	72.4	21	14.1	6.1

**Table 4:** Main smoke parameters by cone calorimetry tests.

Specimen	TSR [m^2^ m^−2^]	SEA [m^2^ kg^−1^]	CO Yield [kg kg^−^^1^]	CO_2_ Yield [kg kg^−1^]	CO/CO_2_ Ratio
EVA	1280.2	597.9	0.0383	3.25	0.0118
15% BC low	955.3	383.4	0.0292	2.63	0.0111
20% BC low	869.6	330.3	0.0309	2.62	0.0498
40% BC low	995.8	406.5	0.0613	2.44	0.0251
15% BC med	1275.6	516.6	0.0332	2.72	0.0122
20% BC med	1240.0	441.1	0.0316	2.39	0.0132
40% BC med	1076.1	508.3	0.0376	2.52	0.0149
15% BC high	1140.5	480.9	0.0445	3.03	0.0147
20% BC high	967.3	373.9	0.0363	2.62	0.0139
40% BC high	989.2	423.8	0.0322	2.39	0.0135

**Table 5:** Tensile properties of EVA and EVA/BC compounds.

Sample	Tensile Modulus (E) [MPa]	Elongation at Break (ε) [%]	Tensile Strength (σ_y_) [MPa]
EVA	50 ± 2.5	123 ± 6.1	6.1 ± 0.1
15% BC low	85 ± 3.7	77 ± 7.2	5.1 ± 0.2
20% BC low	100 ± 2.9	58 ± 5.5	5.3 ± 0.3
40% BC low	211 ± 10.9	26 ± 2.4	8.8 ± 0.4
15% BC med	104 ± 3.2	102 ± 4.1	9.2 ± 0.1
20% BC med	120 ± 1.6	75 ± 3.9	8.9 ± 0.1
40% BC med	212 ± 28.1	24 ± 5.5	7.9 ± 0.7
15% BC high	73 ± 2.6	92 ± 8.3	5.2 ± 0.1
20% BC high	86 ± 3.6	87 ± 3.0	5.1 ± 0.2
40% BC high	221 ± 23.8	24 ± 1.3	8.5 ± 0.6

## Supplementary Materials

The following are available online at https://www.mdpi.com/article/10.3390/polym13081256/s1, Figure S1: screw and temperature profile of the instrument used during extrusion process (KB = kneading blocks), Figure S2: SEM images at 3000× magnification with the corresponding EDX spectra of BC low (A), BC medium (B) and BC high (C), Figure S3: residues after cone calorimetry tests of unfilled EVA (A) and EVA containing 20 wt.% of BC low (B), BC medium (C) and BC high (D), Table S1: results from thermogravimetric analyses in nitrogen and in air for BC powders, Table S2: results from thermogravimetric analyses in air and in nitrogen for EVA and its compounds; Table S3: average results of vertical burning tests for EVA and EVA/BC specimens.
