# Supplementary material for: Investigation of Different Types of Biochar on the Thermal Stability and Fire Retardance of Ethylene-Vinyl Acetate Copolymers
Source: Polymers (Basel). 2021 Apr 13;13(8):1256. doi: 10.3390/polym13081256 (PMC8070515; doi:10.3390/polym13081256)
Supplement: Supplementary file 1 [file polymers-13-01256-s001.pdf]

Supplementary Material

Investigation of different types of biochar on the thermal stability and fire retardance of ethylene-vinyl acetate copolymers

Samuele Matta, Mattia Bartoli, Alberto Frache and Giulio Malucelli

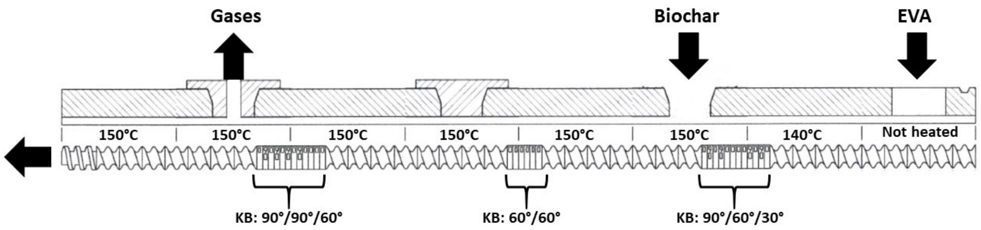

Figure S1: screw and temperature profile of the instrument used during extrusion process (KB = kneading blocks).

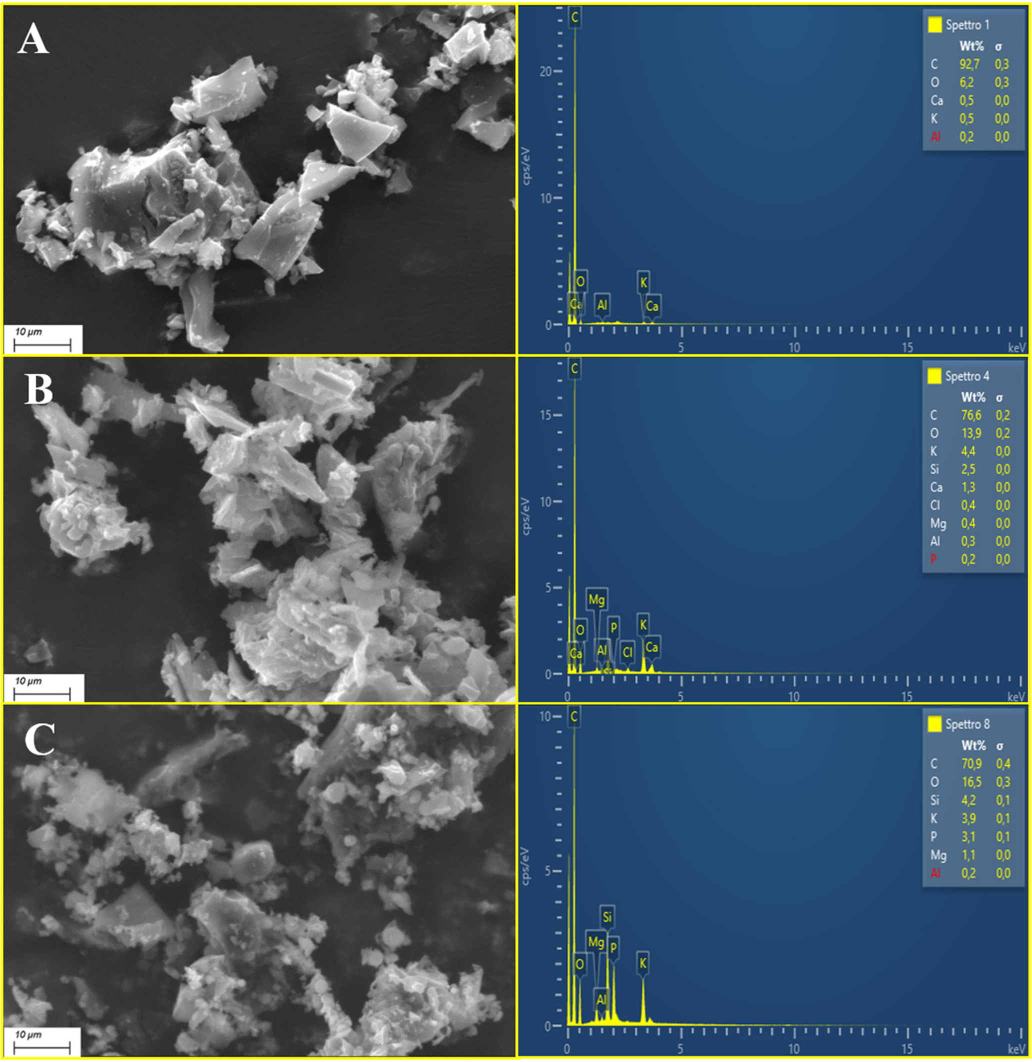

Figure S2: SEM images at 3000 X magnification with the corresponding EDX spectra of BC low (A), BC medium (B) and BC high (C)

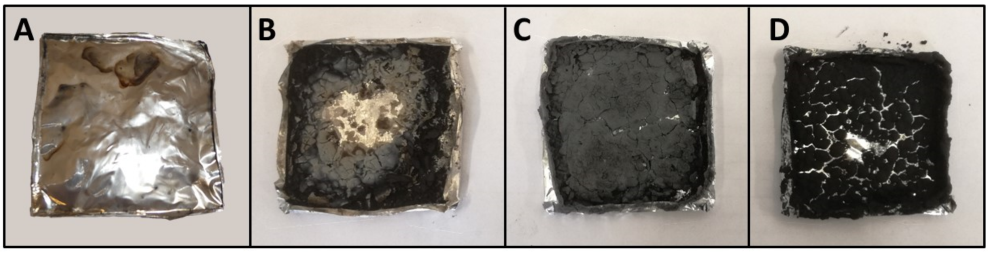

Figure S3: residues after cone calorimetry tests of unfilled EVA (**A**) and EVA containing 20 wt.% of BC low (**B**), BC medium (**C**) and BC high (**D**).

Table S1: results from thermogravimetric analyses in nitrogen and in air for BC powders.

Atmosphere: nitrogen				
**Material**	**T_{onset} [°C]**	**T_{max} [°C]**	**Residue @ T_{max} [°C]**	**Residue @ 700°C [%]**
BC low	-	-	-	90.9
BC medium	-	-	-	88.3
BC high	-	-	-	86.0
Atmosphere: air				
**Material**	**T_{onset} [°C]**	**T_{max} [°C]**	**Residue @ T_{max} [°C]**	**Residue @ 700°C [%]**
BC low	513	597.8	37.0	7.4
BC medium	365	430.4	61.9	24.6
BC high	487	576.9	60.3	39.6

Table S2: results from thermogravimetric analyses in air and in nitrogen for EVA and its compounds.

Atmosphere: nitrogen						
**Sample**	**T_{onset} [°C]**	**T_{max1} [°C]**	**Residue @ T_{max1} [%]**	**T_{max2} [°C]**	**Residue @ T_{max2} [%]**	**Residue @ 700°C [%]**
EVA	322.5	352.8	91.9	471.6	32.1	0.3
15% BC low	328.3	350.8	93.6	471.6	27.1	13.4
20% BC low	328.5	352.9	92.4	472.6	43.4	16.0
40% BC low	327.8	355.1	93.0	468.0	60.2	35.3
15% BC med	325.3	353.7	91.8	475.2	41.6	11.0
20% BC med	325.8	353.4	91.7	464.7	52.4	11.8
40% BC med	334.3	345.8	93.3	476.8	50.3	35.6
15% BC high	326.1	355.9	92.7	471.5	42.3	12.4
20% BC high	327.5	353.7	91.9	471.8	43.1	14.7
40% BC high	332.9	355.0	92.0	475.8	48.1	29.7
Atmosphere: air						
**Sample**	**T_{onset} [°C]**	**T_{max1} [°C]**	**Residue @ T_{max1} [%]**	**T_{max2} [°C]**	**Residue @ T_{max2} [%]**	**Residue @ 700°C [%]**
EVA	304.1	336.9	88.5	431.2	46.9	0.2
15% BC low	307.6	337.1	88.9	432.7	59.6	0.4
20% BC low	310.7	343.5	87.7	438.9	59.3	0.4

40% BC low	322.3	349.4	91.0	452.5	62.5	0.6
15% BC med	319.5	353.7	87.4	475.2	41.6	3.3
20% BC med	320.4	341.1	92.1	475.3	42.8	3.8
40% BC med	327.8	353.5	89.8	475.6	43.0	7.7
15% BC high	308.9	346.6	87.6	430.5	57.8	5.0
20% BC high	312.0	344.2	89.3	453.1	36.7	6.7
40%BC high	318.5	346.4	89.6	467.1	42.5	14.1

Table S3: average results of vertical burning tests for EVA and EVA/BC specimens.

Sample	t_1 [s]	t_2 [s]	Classification
EVA	12	24	V-2
15% BC low	4	4	V-2
20% BC low	6	10	V-2
40% BC low	12	-	NC
15% BC med	7	2	V-2
20% BC med	10	8	V-2
40% BC med	10	-	NC
15% BC high	8	4	V-2
20% BC high	8	9	V-2
40% BC high	14	-	NC
